# Child Mental Health in HIV-Impacted Low-Resource Settings in Developing Countries-Global Research Fellowship: A Research Training Program Protocol

**DOI:** 10.3389/fpubh.2021.632800

**Published:** 2021-04-01

**Authors:** Fred M. Ssewamala, Ozge Sensoy Bahar, Noeline Nakasujja, Betsy Abente, Proscovia Nabunya, Laura Peer, Lily Zmachinski, Suzanne Fragale, Mary M. McKay

**Affiliations:** ^1^Brown School, Washington University in St. Louis, St. Louis, MO, United States; ^2^Department of Psychiatry, College of Health Sciences, Makerere University, Kampala, Uganda

**Keywords:** mental health, HIV, training, Sub-Saharan Africa, research capacity

## Abstract

**Background:** Uganda has one of the highest HIV/AIDS rates and poor mental health services. Children and adolescents in communities with persistent poverty, disease (including HIV/AIDS), and violence, are more likely to suffer from chronic mental health problems. Combined, these characteristics negatively impact communities' response to HIV and mental health beginning with children, adolescents, and young adults. Yet, there is limited research capacity in child and adolescent mental health (CAMH), especially in the HIV/AIDS context in Uganda. Hence, this NIH-funded research training program aims to: (1) train three cohorts of early-career investigators at universities or research institutions in Uganda; (2) connect fellows with committed mentors; and (3) define key factors for successful mentorship and training of new investigators.

**Methods:** CHILD-GRF is a multi-component program that engages selected young investigators in year-round activities for 3 years. Paired with mentors from Washington University in St. Louis and academic institutions in Uganda, fellows participate in a 6-week intensive summer training each year. Year 1 focuses on didactic learning and mentorship. In Year 2, fellows design and conduct their pilot study. Year 3 is devoted to presenting pilot study findings, manuscript preparation/ submission and extramural grant writing.

**Discussion:** CHILD-GRF seeks to provide a solid foundation for the development and implementation of evidence-based HIV prevention and mental health interventions for youth and families impacted by HIV/AIDS. By producing a sustainable network of well-trained individuals in key research institutions, this program contributes to improving CAMH and HIV prevention efforts, both of which have public health implications.

## Introduction

Sub-Saharan Africa (SSA) remains the world's most affected region in the HIV epidemic, home to 71% of people living with HIV worldwide (1). The epidemic continues to spread in young people, with adolescents being reported as the only age group where HIV prevalence is rising (2). Uganda is a SSA country with one of the highest HIV/AIDS rates and poor mental health services (1). Uganda has been affected by high rates of HIV/AIDS (among ages 15–49), especially in large cities, towns and HIV “hot spots.” Kampala, Uganda's capital, has a prevalence rate of 7.1% (3) with the Masaka region reporting a rate of up to 10.6% (1, 3). Uganda also reports poor youth mental health services, a dearth of child and adolescent mental health (CAMH) researchers, community violence, and pervasive poverty (4–7).

Combined, these characteristics negatively impact communities' mental health functioning and response to HIV beginning with children, adolescents and young adults (4, 5, 8). Studies indicate that AIDS-affected communities and family members often suffer recurrent mental health complications including trauma and depression (9–11). This negatively impacts overall HIV care and prevention efforts; and it may severely undermine children's and adolescents' development, social functioning, and reproductive health choices, undermining the overall growth and development of SSA for generations (12).

HIV/AIDS and co-occurring mental health problems are further exacerbated by pervasive poverty (13, 14). Poverty not only affects a community's ability to care for its members' physical and mental health, but also impacts individual members' functioning, and psychosocial well-being (15, 16). Thus, persistent poverty constitutes a critically important risk factor for chronic poor mental health functioning. Specifically, children and adolescents in communities with persistent poverty, disease (including HIV/AIDS), and violence, are more likely to suffer from chronic mental health problems (17). Few efforts in SSA have explored interventions capable of impacting the root causes and consequences of persistent poverty, violence, and co-occurring mental health problems. This is largely due to a lack of infrastructure, capacity to facilitate training and development of research projects, and limited training opportunities for the next generation of a culturally-conscious cadre of researchers in SSA.

In addition, few studies aimed at enhancing mental health functioning of families and communities (and conducted by investigators from SSA), have addressed the risk factors of persistent family poverty, community violence, and HIV/AIDS. Most investigators in SSA target intervention studies that use personal/individual trait models and emphasize generic psychosocial counseling (18). Such interventions often fall short of contextually-grounded, multidisciplinary, combined approaches necessary to break the persistent cycle of poverty, community violence, HIV risk, and mental health challenges. Moreover, it is critical that mental health and HIV prevention interventions in SSA are guided by contextually relevant methods and conceptual models developed and tested in SSA.

Thus, building research capacity that is culturally and contextually responsive to the SSA context in addressing HIV prevention and co-occurring mental health problems in Uganda—a SSA country heavily impacted by HIV/AIDS is an urgent priority. An important step in this direction is a research training program focused on combination interventions addressing persistent poverty, co-occurring mental health problems and HIV infection–all major issues in Uganda, and applicable across most countries in the SSA region. In this paper, we describe the NIH-funded “**C**hild Mental **H**ealth in H**I**V-impacted **L**ow-resource settings in **D**eveloping countries: **G**lobal **R**esearch **F**ellowship” (CHILD-GRF) program (D43TW011541) that provides state-of-the-art methods training, mentoring, and “hands-on” research experience to promising early career research scholars in Uganda committed to research careers focused on addressing the serious CAMH burden in the context of HIV/AIDS. Specifically, the program is designed to achieve the following primary aims:

Identify and train a cadre of 18 SSA scientists from Uganda capable of serving as a Principal Investigators (PIs) on extramurally funded intervention studies focused on combination HIV prevention addressing persistent poverty, community violence, co-occurring child and adolescent mental health problems, and HIV care and prevention in HIV-impacted communities.

Bring together a network of committed mentors from the global north and the global south to ensure quality training for promising new investigators from Uganda who would focus their research on culturally-congruent interventions addressing HIV/AIDS with an emphasis on co-existing CAMH and combination interventions with potential implications for LMICs, including SSA.

Delineate key factors that underlie successful mentorship and training of new investigators from Uganda—with potential implications for new investigators from other SSA countries—who are focused on CAMH and HIV prevention interventions in LMICs, including SSA.

## Background

### Burden of HIV/AIDS Among SSA Youth and Families

Uganda ranks number 11 among SSA countries heavily impacted by HIV/AIDS (19). Adolescents are one of the vulnerable groups where HIV prevalence is rising (2). Adolescence is a particularly vulnerable developmental stage with high risk for HIV, STIs and poor mental health functioning. Higher depression among young people has been associated with co-factors of HIV risk (20). Adolescents facing adversity, particularly poverty, exhibit high rates of early and risky sexual behavior, pregnancy, and drug abuse (21–23). Adolescents affected by AIDS experience even more severe mental health problems than other at-risk groups (24–26). Studies of AIDS-affected Ugandan adolescents living in poverty show high rates of depression (26–28), anxiety, learning problems (29, 30), and risky sexual behaviors (31, 32). Compounded by poverty, the lack of self-esteem and hope for the future can influence sexual risk taking and increase HIV risk. This heightened risk indicates the urgent need for culturally-relevant interventions and a training infrastructure for new researchers. The CHILD-GRF offers fellows the opportunity to study these issues.

### Role of Combination Interventions in Addressing the Burden of HIV/AIDS

Studies and theory suggest causal pathways between family economic resources, education, mental health, and HIV risk (33, 34). Single interventions are often insufficient to address problems as complex and multi-dimensional as CAMH and HIV risk for young people. Hence, investments in combination interventions are critical to provide an interdisciplinary, multi-level response needed to reduce new HIV and STI infections in a way that single interventions alone have not yet been able. UNAIDS and others in the HIV research community have observed an urgent need to shift toward Combination HIV Prevention (CHP) (35–37). CHP seeks to realign program components for maximum effect, to tailor prevention efforts to local epidemics, and to ensure that components are delivered with the intensity, quality, and scale necessary to achieve intended effects (35). Hence, training early stage research scholars in CHP in SSA will strengthen the region's capacity to address the HIV/AIDS burden informed by the rigorous evidence base created by SSA scholars.

### Lack of Culturally-Congruent Prevention Intervention Models

Although advances have been made in the science of CAMH, and HIV intervention and prevention research, the application of scientifically-based, culturally-congruent prevention intervention models tailored to communities heavily affected by poor mental health and HIV remains limited (38). Most HIV prevention interventions for SSA are still guided by theoretical models originated in the global north and lack cultural congruency, and sometimes contextual relevancy (39). The continued spread of HIV within SSA suggests a scientific, moral and ethical imperative for culturally-congruent prevention models that expand upon and/or augment the current western-focused prevention interventions, and incorporate SSA culturally-specific interventions (40).

To date, most HIV intervention programs have been based on theories that are typically social cognitive in orientation: Health Beliefs Model (41–43), Theory of Reasoned Action and Planned Behavior (44–46), Self- efficacy theory (47), and Social Psychological Model (48). These theories emphasize individuals' deficits and few simultaneously attempt to address co-occurring mental health challenges. They also fail to account for the contextual and structural specific issues that influence HIV risk. That may explain the limited success of HIV interventions in SSA that is dominated by structural challenges. In such communities, applying CHP approaches rooted within structural-level theories, such as Ecological perspective (49), Institutional theory (50), Empowerment theory (51), and an Afro-centric paradigm (52, 53) that emphasize structural and culturally specific factors in establishing and maintaining protective behaviors may be needed.

### Need for Capacity Building for Early Career Researchers in SSA

A major barrier to improved HIV prevention, care and treatment is the lack of capacity to conduct locally relevant research, often due to the limited number of scientists and health professionals in low- and middle-income countries (LMICs) equipped with the necessary research expertise and training (54). A recent Lancet report on authorship found that only 35% of publications addressing research interventions in LMICs, include authors from LMIC settings (55). This points to the need for additional collaborations, and further strengthening research and dissemination skills of promising in-country early stage investigators. It is also important to note that to-date, very few scholars in SSA have been able to compete for extramural funding, primarily due to a lack of infrastructure and investment in training the next generation of investigators.

## Description of Child-GRF Training Program

CHILD-GRF, currently in Year 1, is a collaborative multi-component training program that aims to prepare young investigators for year-round activities to focus their research on serious overlapping CAMH outcomes experienced by HIV-impacted youth, families and communities, particularly in the context of persistent poverty. These activities include in-person and on-line seminars, webinars and workshops; regular mentorship meetings; social networking events; and hands-on practical research experience. The program includes face-to-face intensive training and mentoring by faculty at Washington University in St. Louis (WashU) for three summers. The training is designed to ensure fellows establish a strong foundation to secure extramural funds to conduct culturally-congruent rigorous HIV prevention and CAMH intervention, services and Dissemination & Implementation (D&I) research within SSA; and to provide collective mentorship to all fellows.

### Training Program Structure

Each spring, CHILD-GRT enrolls six fellows per year in the first 3 years for a 3-year training (18 fellows total over the 5-year program period). CHILD-GRF consists of year-round activities and pilot study funding awarded in the second year to generate preliminary data that will support proposals for larger, extramurally funded research. The program also has a 6-week intensive summer session (18 weeks total over a 3-year period) focused on developing skills needed to collaboratively develop, implement, test, disseminate and scale evidence-informed, culturally-congruent HIV prevention, and CAMH interventions. Throughout the 3 years, intensive research training is offered every summer by experts in HIV prevention, CAMH services research and combination interventions that incorporate family economic strengthening, psychosocial and biomedical interventions. In addition, fellows will draft a research grant application to be submitted for extramural funding. Upon completion of the 3-year program, each CHILD-GRF fellow will receive a program certificate.

#### Core Curriculum for Summer Intensive Training Component

The curriculum is comprised of a 6-week yearly summer intensive training. Curriculum for the first year is delivered by faculty with expertise in HIV prevention, CAMH services, and combination interventions. In addition to coursework during the second summer, fellows are expected to present a designed/drafted pilot study proposal developed with feedback from their mentors to be reviewed and critiqued by a peer review committee comprised of at least three reviewers from the Executive Committee (EC)/Training Advisory Committee (TAC)/Mentorship Committee and one fellow (a peer). The third-year summer curriculum is devoted to: (1) presentation of findings from the fellows' funded pilot studies; (2) training mentees in manuscript preparation/submission; and (3) extramural grant writing. Each fellow works with their mentors to develop and implement individual projects.

Additionally, fellows are required to attend mentoring sessions and advanced seminar series providing opportunities to share the research design of their projects with their colleagues and mentors for feedback. After the third-year summer meeting, additional support will be facilitated via webinars and distance learning technology. In the final year of the program, Year 5, all cohorts (*N* = 18 fellows) will attend a CHILD-GRF networking meeting/conference organized by the CHILD-GRF leadership at WashU where fellows will present their work and plan future collaborations (see Table 1 for Year 1's curriculum and Table 2 for an overview of the planned curriculum for Years 2 and 3).

**Table 1:** First year intensive summer training curriculum by subject.

**Research methods**
The importance of adaptive and tailored approaches to implementation in addressing dynamic challenges
Agent-based modeling
Applications of qualitative methods in research
Community based system dynamics
Evaluation planning
Community collaboration in research
**Global research topics**
Domestic and global health disparities
Research challenges, opportunities in Sub-Saharan Africa during COVID-19
Systemic racism and mental health
Global mental health, parts I & II
HIV and global mental health priorities & funding opportunities & guidance
The potential of economic strengthening interventions in the care and support of children and adolescents impacted by HIV/AIDS: lessons from 15 years of research in Sub-Saharan Africa
Research to eliminate neglected tropical diseases
Mental health interventions in Uganda
**Data collection, management, & dissemination**
Categorical data methods and logistic regression
Utilization & dissemination of results
Cost effectiveness
Propensity score analysis: an overview & latest debates
Administrative data management and analysis
**Career development**
Manuscript development
NIMHD mission and funding opportunities
Leaky pipeline/women and minorities in research
How to give a presentation
More than spoken words: race and racism and the implications for scientific methods and researcher resiliency
The psychology of mindset and resilience
Responsible conduct of research workshop
A clinical research career
Career panel and closing session

**Table 2:** Overview of the intensive summer training courses in years 2 and 3.

**Intensive summer courses II (duration: 6 weeks year 2)**	**Intensive summer courses III (Duration: 6 weeks year 3)**
Child & adolescent development	Grant writing workshops
Social determinants of health/mental health	Manuscript writing workshops
Advanced topics in statistics	
Health psychology and health education models	
Health economics	
Community-Based participatory research	
Designing combination interventions	
Dissemination & implementation research	
Scientific integrity & ethical conduct of research refresher	
Career development workshop	
Policy analysis	
Child mental health in humanitarian crisis situations	
Evidence-based practice and cultural adaptation	

The 3-year summer intensive training period is intended to give fellows enough time to prepare strong and competitive applications. Moreover, this program design allows us to at least have two cohorts of fellows interact at any one time (3 cohorts interact at summer training in Years 3 and 5), share experiences, and form an HIV prevention researcher network for future collaborations.

#### COVID-19-Related Adaptations to the Summer Intensive Training

Due to COVID-19, the CHILD-GRF curriculum shifted from a 6-week in-person training at WashU to an online 10-week series of didactic training, program cohort meetings, and meetings with mentors. The online format allowed us to invite guest lecturers from across the US and Uganda, resulting in a wider range of topics and perspectives. This format also enabled us to be responsive to feedback from the fellows in real time; and incorporate additional research methodology content and opportunities to discuss current event topics such as the COVID-19 pandemic. Through the online platform, we incorporated the CHILD-GRF program into our two other NIH-funded mental health-focused training programs (R25MH118935; T37MD014218) that ran concurrently. By combining all three training programs, we leveraged our resources and created opportunities for more networking, bi-directional peer feedback, and support from a larger cohort of fellows and faculty mentors. Moreover, CHILD-GRF fellows had the opportunity to participate in an NIH mock review session as observers and peer reviewers for pilot grant applications from R25 Researcher Resilience Training Program fellows. This allowed them to gain valuable insight into the NIH review process and practice their own review skills as reviewers during the summer training period. CHILD-GRF fellows concluded their first summer training by giving final presentations on work accomplished and next steps. The fellows continue to work with their mentors on their individual research projects.

#### Independent Research Projects and Pilot Seed Funding

Following the fellows' second year of summer training, research seed funds are available. Fellows submit a 6-page proposal with specific aims, research strategy, data analysis plan and protection of Human Subjects sections. Applications are reviewed by the EC that provides feedback and guidance before funding is dispersed. No funding is disbursed without a fellow having Institutional Review Board approval. This funding is critical for fellows to establish the groundwork and preliminary/pilot data to inform extramural grant application submissions.

#### Mentoring Model

The CHILD-GRF program has mentorship trainings for new mentors annually. This 2-h workshop provides an overview of mentorship including: the phases of mentorship; establishing expectations; effective communication techniques; how to develop a successful individual development plans with your mentees; and characteristics of successful and unsuccessful mentorships. It also allows for dialogue between mentors to discuss best practices and troubleshoot areas where mentors may be experiencing difficulties.

**Figure 1:**
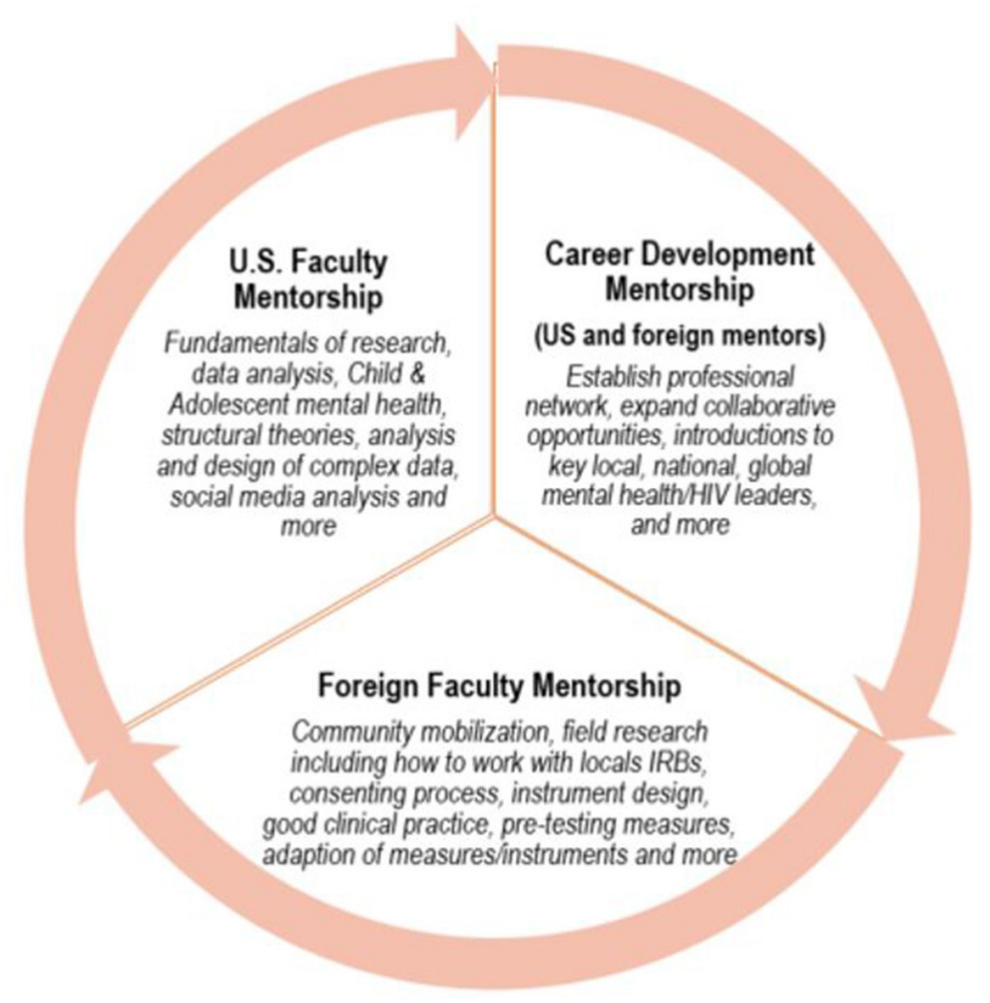
Mentorship model.

Fellows receive direct mentorship from two faculty members, one US-based and one based in Uganda (see Figure 1). A mentor-mentee agreement is signed to lay out expectations, and roles and responsibilities of their relationship. During the initial 6 weeks in St. Louis, fellows meet with their assigned WashU faculty member who provides regular long-distance mentorship and guidance to submit conference abstracts, grants and/or manuscripts during the 3-year program. Starting in the second week of the program, fellows meet virtually twice per week to work directly with their local in-country mentors.

Over the course of the 3-year program, WashU and in-country mentors are expected to meet virtually for an hour every other week to review fellows' progress and work through any issues. An Individual Development Plan (IDP) will be drafted and signed by both mentors and the mentee. Mentors are also expected to remain in touch with fellows either in-person or by telephone, email, or virtual meetings with the goal of supporting mentees to submit conference abstracts, grants and/or manuscripts.

**Figure 2:**
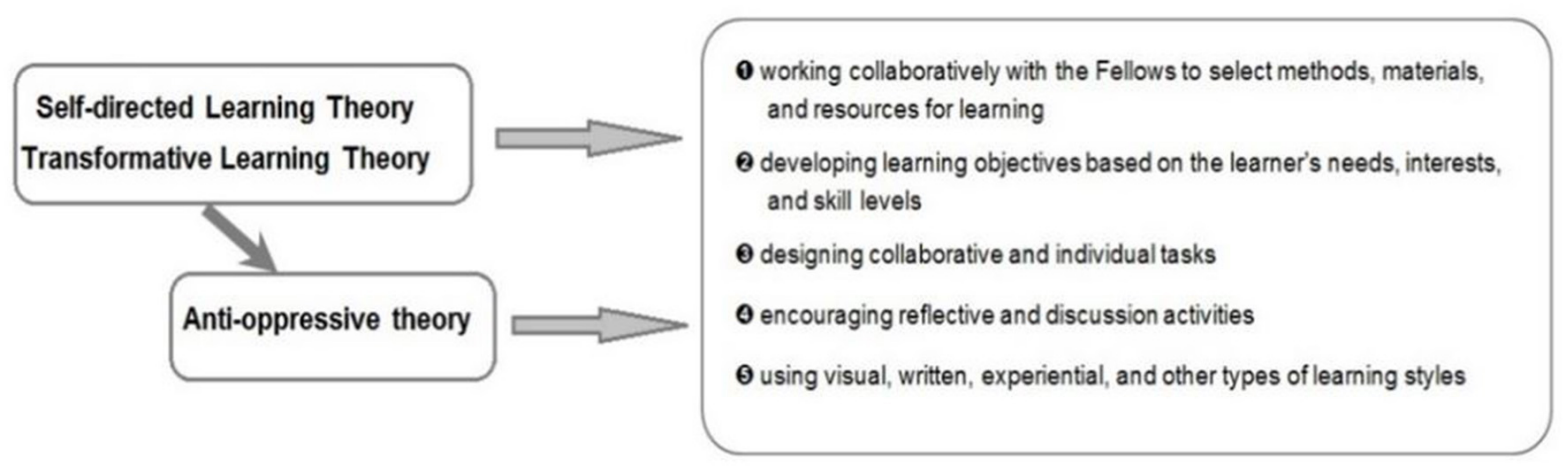
Learning theories.

To address varied learning styles and cultures of education, the mentoring model is informed by adult learning theories: Self-directed learning (56–58), and Transformative learning (59, 60) theories. In addition, mentoring activities are informed by anti-oppressive theory (61–63), to create a culturally-sensitive, contextually-relevant, and inclusive learning environment (see Figure 2). This is achieved by ensuring a learning environment that balances different learning styles and cultures through: (1) collaborating to select methods, materials, and resources; (2) developing learning objectives based on each learner's needs, interests, and skill levels; (3) designing collaborative and individual tasks; (4) encouraging reflective and discussion activities; (5) using visual, written, experiential, and other types of learning styles; and (6) evaluating and adjusting the quality of the learning experience while assessing further learning needs. The anti-oppressive theory, focused on targeting structural inequalities and minimizing power hierarchies (61–63), is particularly relevant to guide CHILD-GRF's goal to invest in building capacity among early career researchers to address inequity in global north-south research collaborations. Researchers from the global north can have a utilitarian approach in north-south research collaborations with less focus on sustainable capacity building (64). These relationships can become skewed and driven entirely by research output for global north researchers and short-term financial incentives and development of static power hubs for global south researchers. For research to be led by global south researchers and to allow significant leadership to develop, investing resources in capacity building, leadership and local development in science is critical. CHILD-GRF is a critical step toward this goal.

Mentor pairing decisions are made by the EC based on areas of interest, as well as individuals' learning needs. The EC draws on information provided in the fellow's application and their individualized learning and career development plans. During the 3-year program, mentors and mentees fill out electronic evaluation forms at the end of each summer to evaluate their relationship. These surveys assess each mentor's time commitment, availability, and effectiveness; and determine specific problems and strengths of the relationship. If problems arise, the Program Directors will meet with both parties and if necessary, propose a new mentor.

### CHILD-GRF Application Selection

#### Participants

Three cohorts of early career investigators committed to conducting HIV prevention and CAMH intervention, and D&I research within resource constrained communities in Uganda, will be recruited (*n* = 18 fellows over a 3-year period). Candidate selection is conducted by the Selection Committee (comprised of the EC detailed above) based on the following selection criteria:

1. Candidates must be early career researchers (advanced PhD students, recent PhD or MD graduates working as junior faculty or researchers at a recognized and accredited academic or research institution in Uganda).
2. Candidates must demonstrate a track record of excellence and the potential for a successful research trajectory in academia.
3. Candidates must submit two letters of recommendation from faculty/senior research scientists who have first-hand knowledge of their potential for conducting research related to HIV prevention or CAMH in communities impacted by HIV/AIDS.
4. All candidates must submit personal statements indicating their interest in HIV prevention or CAMH in communities heavily impacted by HIV/AIDS.
5. Candidates must submit a signed agreement indicating they will attend all required mentorship and program activities.
6. A letter from the department chair, or equivalent, should be submitted, stating the fellow will: (a) have access to necessary resources in their institution enabling them to conduct their research projects; (b) commit at least 30% effort to attend the Intensive Research Courses and all mentoring activities; and (c) be granted time off to attend summer training courses at WashU.

#### Fellow Selection Process

All applications are rated according to the criteria above. Accordingly, applications are assigned to members of the Selection Committee, who then present a critique of the applications during a closed webinar session. After discussion, each application is scored and ranked. Up to 15 top candidates are interviewed (face-to-face or virtually) by the Program Directors/EC. Interviews are designed to ascertain the candidate's research capacity, research interest, and expectations from the training program. The candidates are rated based on these criteria and the top six are selected and notified of acceptance in April. The remaining candidates are put on a waiting list, which can be activated should a selected candidate decline the invitation.

### Administrative Structure

CHILD-GRF is led by a multidisciplinary team of accomplished investigators from both the USA and Uganda. The overall program is co-led by a team comprising of US-based and Uganda-based researchers. Leadership is supported by a strong team of EC members that will be responsible for high-level oversight and implementation of the program. The EC is supported by a Training Advisory Committee (TAC) which provides strategic and technical oversight (see Table 3).

**Table 3:** Roles and responsibilities of EC and TAC.

**Executive committee**	**Training advisory committee**
Oversight: Administer the program, including general oversight of the program and grant management, planning, and executing decisions regarding program activities	Recruitment: Assistance with recruitment and retention of potential trainees and mentors. Help develop a trainee “pipeline”
Trainee selection: Select candidates for the program	Review: Examine evaluation reports of didactic sessions/workshops and provide guidance on implementation of appropriate recommendations
Mentor selection and training: Match candidates with appropriate Faculty Mentors and provide guidance to selected mentors	Cultural relevance: Review cultural relevance and sustainability of the training program and how best to incorporate cultural relevant topics in the training
Quality control: Ensure that trainees receive adequate mentorship and research opportunities	Recommendation: Recommend program innovations and improvements to the EC including development of a sustainable infrastructure
Course correction: Modify the training needs based on TAC recommendations	
Evaluation: Evaluate the development progress of trainees, assess the impact of each of the program activities, and elucidate factors associated with greater or lesser success	

### Evaluation Plan

Fellows' data are systematically collected via IDPs which are jointly completed by each fellow and mentor at the beginning of the program and revisited at least bi-annually. This standardized form tracks the fellow's disposition of submitted manuscripts; research and career development milestones, frequency, and duration of mentor meetings; program activity participation; and any other career development endeavors. Fellows' CVs are also collected for 15 years, allowing for longer-term assessment of research milestones (e.g., publications). More specifically, the evaluation materials include the following:

1. Course evaluations: Courses are evaluated via direct observation by Program Directors and written evaluations from fellows and mentors. Evaluation questions assess clarity of expectations, organization and content of lectures and materials, topic relevance, instructor engagement, encouragement of critical thinking, and global assessment of the instructor. Each training webinar/lecture/workshop is followed by a brief evaluation that includes a space for suggestions for future topics. Surveys are also sent out at the end of each summer program to receive feedback on different components of the program, including speakers, course content, mentorship. In addition to the surveys -that include open-ended questions, semi-structured interviews are conducted with each fellow at the end of their participation in the 3-year program as part of their exit interviews. These interviews are intended to gain more in-depth understanding of their experience with the CHILD-GRF program, including courses, mentorship, hands-on learning experience, their independent studies, as well as barriers and facilitators to participation. Although the small number of fellows most likely prohibits statistical inference, qualitative data collected is used to gain insights into predictors of success and areas of improvement. In short, surveys, qualitative interviews, and exit interviews are used to evaluate quality, accessibility, coherence, additional research topics, and access to research support.
2. Mentor evaluations: At the middle and end of the summer program, fellows complete online surveys to evaluate their mentoring relationship. These surveys assess each mentor's time commitment, availability, and effectiveness as well as determine specific problems and strengths of the relationship.
3. Fellow evaluations: Mentors evaluate fellows at the end of the summer program using online surveys to identify possible problems and redirect scholar efforts when necessary. Mentor feedback also addresses knowledge, skills, critical thinking, drive, productivity, creativity, interpersonal skills, and presentation skills. This feedback is shared with TAC members and fellows to help guide career development.
4. Fellow tracking: Fellows are tracked for 15 years to obtain long-term feedback and insights on program benefits, pitfalls and effectiveness. Fellow employment, career development, and milestones, including research positions, faculty appointments, grants awarded, presentations, publications, awards, participation in study sections, and mentoring record are monitored.
5. Bibliometric analysis: CHILD-GRF Program Directors use social network analysis to assess scholarly collaboration to research productivity.

Fellows' progress in manuscript preparation and grant submissions will be tracked and submitted annually to the EC. The EC takes these results, and with input from the TAC, identifies areas needed to be strengthened or further developed.

## Discussion

Research training programs in HIV prevention remain of critical importance to generate evidence that may save lives, especially in LMICs, including SSA countries with the biggest disease burden. CHILD-GRF's goal is to support the development of a cadre of young researchers working in countries heavily impacted by HIV/AIDS to conduct CAMH research using rigorous culturally-congruent research methods, and testing combination HIV prevention interventions.

The CHILD-GRF program is innovative in several ways. First, acknowledging that single interventions are useful but insufficient, the CHILD-GRF focuses on Combination HIV Prevention (CHP) approaches, including those that combine economic empowerment, psychosocial and biomedical interventions (where applicable). Program fellows can implement these approaches by learning from and building upon the expertise of the network of mentors and faculty speakers.

Secondly, the program offers an opportunity to address inequity in global north-south research partnerships by building capacity among early career researchers in SSA. Equity implies giving equal opportunities to a diverse set of people with differential capabilities to succeed. In trying to address local problems in a joint research enterprise, global partnerships may foster goodwill and a model of furthering scientific enterprise at the core. Yet, the issue of how benefits, resources, and knowledge are developed and shared continues to be problematic (65, 66). For instance, although much of HIV-related research happens in developing countries, with a majority happening in SSA, the majority of the lead researchers are from the global north, with African researchers being used as data collectors (67). CHILD-GRF fellows are trained and mentored to design and lead interventions that are culturally-relevant and specifically address the burden of HIV infection (2, 68, 69) and heightened mental health stressors in communities in SSA. Offering rigorous training and mentorship for young scholars while fostering partnerships with local research and academic institutions will ultimately increase the research capacity in this region.

Few academic researchers have been trained to establish and maintain satisfactory collaborations with community-based sites. Frequently, communities have felt exploited by researchers who conduct studies without understanding their needs and then leave, giving little in return (67). Researchers need to be creative and innovative in their approaches to community collaborations. Input from community-based institutions should actively be sought during the intervention design phase to ensure maximum “real world” and cultural relevance (70). Community input may increase the likelihood of interventions that may be efficacious in academic environments to be successfully duplicated in real-world settings. Thus, there is a need for effective collaborations between researchers and local institutions. CHILD-GRF emphasizes partnerships between researchers and local institutions by engaging them in the recruitment of fellows; actual training via mentorship and TAC; and local institutions offering their agencies to act as “research implementation labs” with whom the fellows may undertake both the pilot studies and subsequent large-scale studies. This partnership allows the program to capitalize on indigenous community resources in developing culturally-congruent and local policy-relevant research programs by the fellows and their mentors. Moreover, the fellows can learn how to involve the community in all aspects of their research.

In conclusion, the CHILD-GRF has great potential to provide a robust platform for the development and implementation of culturally-congruent evidence-based HIV prevention and CAMH interventions for youth and families living in communities heavily impacted by HIV/AIDS. By producing a sustainable network of individuals in key research institutions who are well-trained in CAMH, HIV/AIDS, and D&I research, this training program will contribute to improving CAMH and HIV prevention efforts, both of which have implications for public health.

## Author Contributions

FMS, OSB, NN, BA, and MM contributed to the conceptualization of the training program. FMS, NN, and MM are multiple principal investigators on the grant. BA, SF, and LP have contributed to the implementation of the program. All authors have contributed to the writing of the manuscript.

## Conflict of Interest

The authors declare that the research was conducted in the absence of any commercial or financial relationships that could be construed as a potential conflict of interest.
